# Supplementary figures and images for: A Genome-Wide Association Analysis Reveals Epistatic Cancellation of Additive Genetic Variance for Root Length in Arabidopsis thaliana
Source: PLoS Genet. 2015 Sep 23;11(9):e1005541. doi: 10.1371/journal.pgen.1005541 (PMC4580642; doi:10.1371/journal.pgen.1005541)

**A**

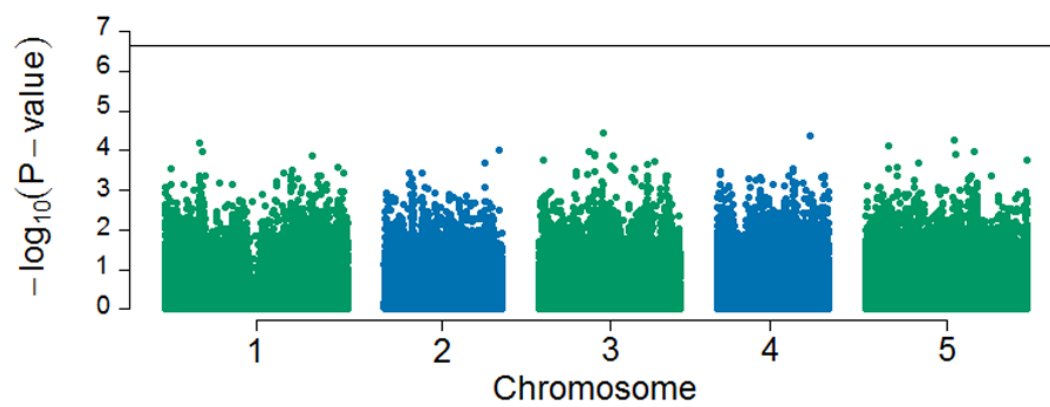

**B**

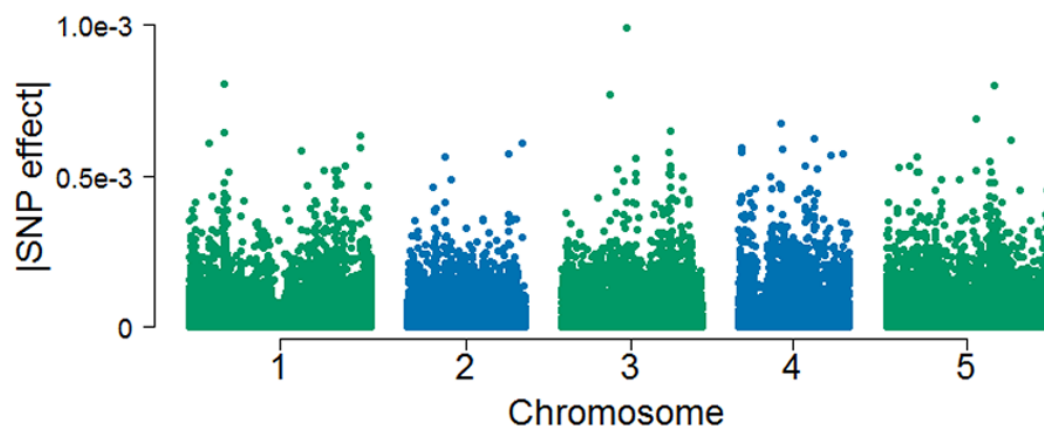

Supplement: S1 Fig — (A) In the GWAS based on an additive model no SNPs reached the Bonferroni threshold. (B) Using a whole-genome generalized ridge regression model, in which SNPs were modeled as random effects, no SNPs reached the Bonferroni threshold. (PDF) [file pgen.1005541.s002.pdf]

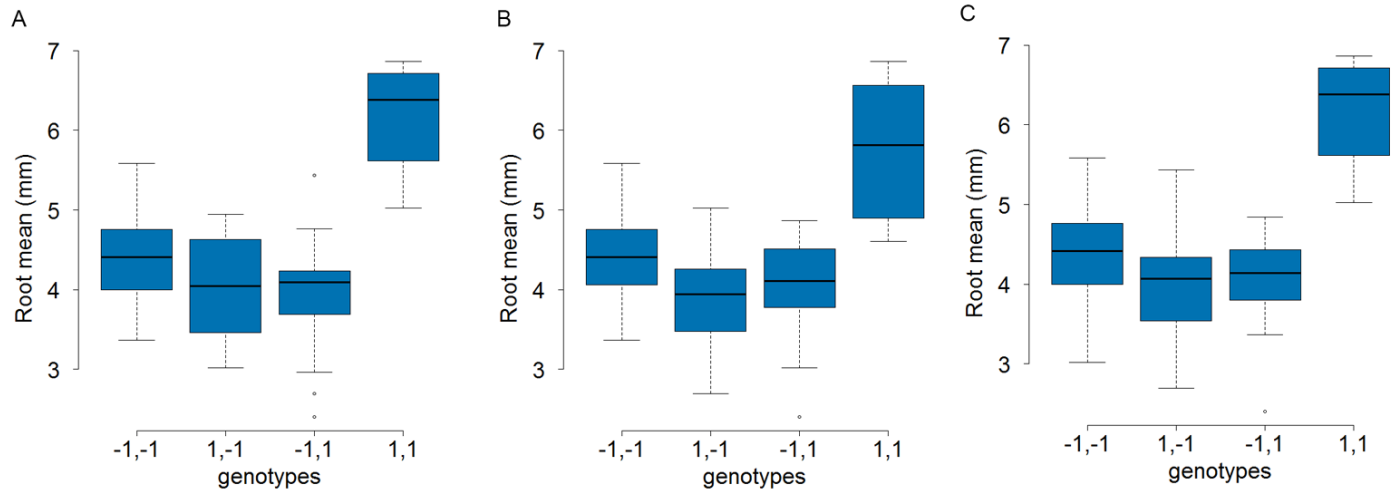

Supplement: S2 Fig — (A-C) Mean root length is displayed for all four two-locus genotype-classes for the four pairs of significantly interacting loci. The major allele is indicated by -1 and the minor allele is indicated by 1. The combination of two minor alleles (1,1) always has the longest root length compared to the other three-allele combinations, which are more similar to one another. Pictured are mean root lengths for the epistatic pairs for SNPs (A) 3_66596 and 3_9272294/3_9273674, (B) 3_66596 and 5_18241640, and (C) 3_10891195 and 5_1027939. (PDF) [file pgen.1005541.s003.pdf]
