# Supplementary material for: A Genome-Wide Association Analysis Reveals Epistatic Cancellation of Additive Genetic Variance for Root Length in Arabidopsis thaliana
Source: PLoS Genet. 2015 Sep 23;11(9):e1005541. doi: 10.1371/journal.pgen.1005541 (PMC4580642; doi:10.1371/journal.pgen.1005541)
Supplement: S1 Table — (DOCX) [file pgen.1005541.s004.docx]

**S1 Table. *A. thaliana* accessions phenotyped for GWA.**

| accession | stock number |  | accession | stock number |  | accession | stock number |
| --- | --- | --- | --- | --- | --- | --- | --- |
| Ag-0 | CS22630 |  | Kin-0 | CS22654 |  | Ren-11 | CS22611 |
| An-1 | CS22626 |  | Knox-10 | CS22411 |  | Rmx-A02 | CS22568 |
| Bay-0 | CS22633 |  | Knox-18 | CS22567 |  | Rmx-A180 | CS22569 |
| Bil-5 | CS22578 |  | Kondara | CS22651 |  | RRS-10 | CS22565 |
| Bor-1 | CS22590 |  | Kz-1 | CS22606 |  | RRS-7 | CS22564 |
| Bor-4 | CS22591 |  | Kz-9 | CS22607 |  | Se-0 | CS22646 |
| Br-0 | CS22628 |  | Ler-1 | CS22618 |  | Shakdara | CS22652 |
| Bur-0 | CS22656 |  | LL-0 | CS22650 |  | Sorbo | CS22653 |
| C24 | CS22620 |  | Löv-1 | CS22574 |  | Spr1-2 | CS22582 |
| CIBC-17 | CS22603 |  | Löv-5 | CS22575 |  | Sq-1 | CS22600 |
| CIBC-5 | CS22602 |  | Lp2-2 | CS22594 |  | Sq-8 | CS22601 |
| Col-0 | CS22625 |  | Lp2-6 | CS22595 |  | Tamm-2 | CS22604 |
| N13 | CS22491 |  | Lz-0 | CS22615 |  | Tamm-27 | CS22605 |
| Ct-1 | CS22639 |  | Mr-0 | CS22640 |  | Ts-1 | CS22647 |
| CVI-0 | CS22614 |  | Mrk-0 | CS22635 |  | Ts-5 | CS22648 |
| Eden-1 | CS22572 |  | Ms-0 | CS22655 |  | Tsu-1 | CS22641 |
| Eden-2 | CS22573 |  | Mt-0 | CS22642 |  | Ull2-3 | CS22587 |
| Edi-0 | CS22657 |  | Mz-0 | CS22636 |  | Ull2-5 | CS22586 |
| Ei-2 | CS22616 |  | Nd-1 | CS22619 |  | Uod-1 | CS22612 |
| Est-1 | CS22629 |  | NFA-10 | CS22599 |  | Uod-7 | CS22613 |
| Fab-2 | CS22576 |  | NFA-8 | CS22598 |  | Van-0 | CS22627 |
| Fab-4 | CS22577 |  | Nok-3 | CS22643 |  | Vår2-1 | CS22580 |
| Fei-0 | CS22645 |  | ÖMö2-1 | CS22584 |  | Vår2-6 | CS22581 |
| Ga-0 | CS22634 |  | ÖMö2-3 | CS22585 |  | Wa-1 | CS22644 |
| Got-22 | CS22609 |  | Oy-0 | CS22658 |  | Wei-0 | CS22622 |
| Got-7 | CS22608 |  | Pna-17 | CS22570 |  | Ws-0 | CS22623 |
| Gu-0 | CS22617 |  | Pro-0 | CS22649 |  | Ws-2 | CS22659 |
| Gy-0 | CS22631 |  | Pu2-23 | CS22593 |  | Wt-5 | CS22637 |
| HR-10 | CS22597 |  | Pu2-7 | CS22592 |  | Yo-0 | CS22624 |
| HR-5 | CS22596 |  | Ra-0 | CS22632 |  | Zdr-1 | CS22588 |
| Kas-2 | CS6751 |  | Ren-1 | CS22610 |  | Zdr-6 | CS22589 |
