# Supplementary material for: A Genome-Wide Association Analysis Reveals Epistatic Cancellation of Additive Genetic Variance for Root Length in Arabidopsis thaliana
Source: PLoS Genet. 2015 Sep 23;11(9):e1005541. doi: 10.1371/journal.pgen.1005541 (PMC4580642; doi:10.1371/journal.pgen.1005541)
Supplement: S2 Table — (DOCX) [file pgen.1005541.s005.docx]

**S2 Table. T-DNA lines tested for root phenotypes.**

| Gene | T-DNA line | Insertion location |
| --- | --- | --- |
| At3g01185 | SALK_033165 | promoter |
| At3g01200 | SALK_035653 | Exon 1 of 3 |
| At3g25520 | SALK_135037 | 5’UTR |
| At3g25530 | SALK_057410 | Intron 3 of 7 |
| At3g25540 | SALK_073240 | Exon 1 of 5 |
| At5g45120 | SALK_030666 | 5’UTR |
| At5g39610 | SALK_090154 | Exon 3 of 3 |
| At5g39620 | SALK_096950 | Intergenic |
| At3g28865 | SALK_089352 | Exon 1 of 1 |
| At3g28865 | SALK_009759 | Exon 1 of 1 |
| At3g28880 | SALK_070443 | Exon 17 of 19 |
| At5g03840 | SALK_142051 | 5’ UTR |
| At5g03850 | SALK_112775 | Exon 1 of 1 |
